# Long-Distance Travel Behaviours Accelerate and Aggravate the Large-Scale Spatial Spreading of Infectious Diseases

**DOI:** 10.1155/2014/295028

**Published:** 2014-01-08

**Authors:** Zhijing Xu, Zhenghu Zu, Tao Zheng, Wendou Zhang, Qing Xu, Jinjie Liu

**Affiliations:** Center for Biosecurity Strategy Management, Beijing Institute of Biotechnology, Beijing 100071, China

## Abstract

The study analyses the role of long-distance travel behaviours on the large-scale spatial spreading of directly transmitted infectious diseases, focusing on two different travel types in terms of the travellers travelling to a specific group or not. For this purpose, we have formulated and analysed a metapopulation model in which the individuals in each subpopulation are organised into a scale-free contact network. The long-distance travellers between the subpopulations will temporarily change the network structure of the destination subpopulation through the “merging effects (MEs),” which indicates that the travellers will be regarded as either connected components or isolated nodes in the contact network. The results show that the presence of the MEs has constantly accelerated the transmission of the diseases and aggravated the outbreaks compared to the scenario in which the diversity of the long-distance travel types is arbitrarily discarded. Sensitivity analyses show that these results are relatively constant regarding a wide range variation of several model parameters. Our study has highlighted several important causes which could significantly affect the spatiotemporal disease dynamics neglected by the present studies.

## 1. Introduction

Modelling the spatial disease dynamics has long been a research priority because of its potential in facilitating the development of mitigation strategies responding to the increasing threat of an influenza pandemic [1, 2]. The large-scale spatiotemporal spreading of diseases transmitted person to person via droplets or direct contact in real population is ultimately denominated by the population interaction which could be described by a social contact network and by interplay with the virus itself [3, 4]. Networks have provided a unified way to think about the daily interaction between individuals, are especially helpful when each individual is in direct contact with only a small proportion of the population [5–9], and thus have been powerful tools for understanding the transmission of infection in the population due to either social contact or sexual contact. Several types of classical networks have been frequently used in epidemiological literatures, which are random networks [10], lattices [11], small world networks [12], spatial networks [13] and scale-free networks [14]. Recently, researchers have incorporated demogeographic information into the contact network and integrated it with metapopulation model based on human mobility data [15–21] to pursue a better understanding of the role of real population structure and human mobility in the large-scale transmission of the diseases. The long-distance travel, which is the primary driving factor for the intersubpopulation pervasion of the contagion [20, 22], has been explored a lot. However, when evaluating the risk of infection for long-distance travellers, present studies have arbitrarily neglected the travel types and assumed all the infectious individuals in targeted population will lead to equivalent risk to the travellers, which results in an underestimation of the risk.

**Figure 1:**
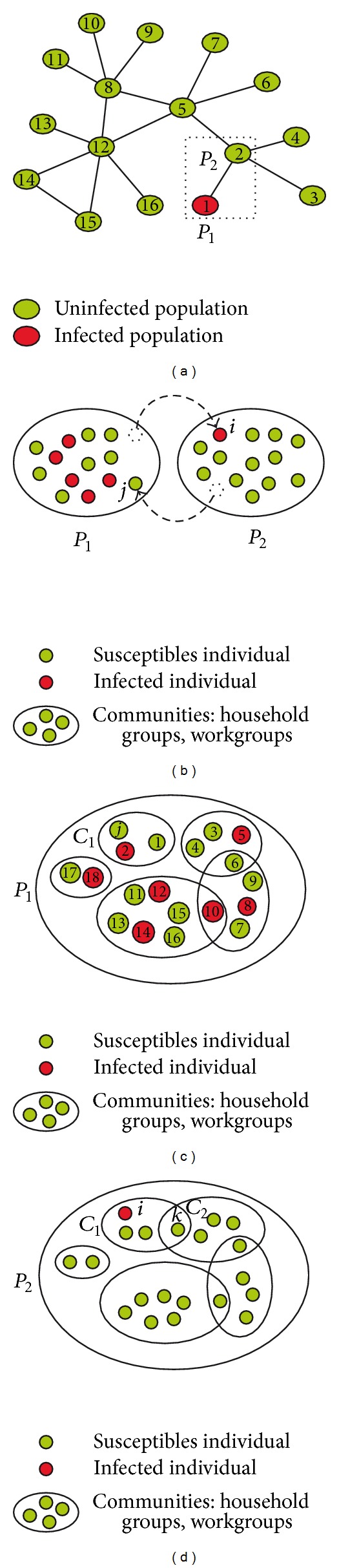
Schematic of metapopulation system. (a) A metapopulation system with several relatively isolated populations. These populations are synchronised by individual flows to some degree but not fully. At the beginning of the simulation, the epidemic outbreaks in population *P*_1_, while other populations stay susceptible. (b) A long-distance traveller *i* travels from *P*_1_ to *P*_2_, and at the same time, another long-distance traveller *j* travels from *P*_2_ to *P*_1_. Thus individuals in *P*_2_ are exposed to the risk of infection. With no MEs, every infective in *P*_1_ leads to equivalent risk to *j*. (c) The population structure in *P*_1_. Individuals are organised into groups and these groups may be overlapped. The long-distance traveller *j* stays in group *C*_1_ when visiting *P*_1_. In this case, *j* is more likely to be infected by 2 than by other infectives. And owing to the higher transmission rate within the groups, *j* will suffer from a higher risk. (d) The population structure in *P*_2_. *i* stays in group *C*_1_ when visiting *P*_2_ and susceptibles in *C*_1_ are more likely to be infected compared to other susceptibles in *P*_2_. The higher risk could then be transferred to group *C*_2_ through *k*. This process is repeated and thus accelerates the spreading of infectious diseases.

In real world, people make their long-distance travel for different purposes, such as tourism, business, and visiting friends and relatives (VFR) [23]. These diverse travel types will lead to corresponding changes to the structure of the destination population, thus affecting the spreading dynamics of the epidemics. Suppose an emerging infectious disease outbreaks in population *P*_1_ and all the other populations stay susceptible (as shown in Figure 1(a)) and *i*, *j* are two VFR travellers. In the spatially explicit simulation approach, in which the population is defined in terms of different groups, VFR traveller *j* will enter into a specific group *C*_1_ and thus will suffer from higher risk from infectives in this group than in the general community, which could be named “merging effects (MEs).” The MEs will result in a different population structure as shown in Figure 1(c) in contrast to that shown in Figure 1(b). The MEs will also take effects for business travellers who would be most likely to enter into a specific workgroup, while taking no effects for tourism travellers.

In this paper we present a metapopulation model with the individuals in each subpopulation organised into a scale-free contact network. The subpopulations are interconnected through two types of the long-distance travel based on whether the travellers are merging into the contact network of the destination subpopulation or not. We then examine the influences of the MEs on the spatiotemporal disease dynamics. The results show that the MEs have constantly accelerated and aggravated the propagating processes of the epidemics and higher merging intensity will strengthen these two effects. The sensitivity analyses for the travel rate show that a higher travel rate leads to more rapid disease transmission, while at the same time it alleviates the outbreaks. The impacts of the return rate are relatively uncertain due to its duplicity—a higher return rate implies higher human mobility yet lower possibility of being infected.

## 2. Methods

### 2.1. Social Contact Networks

In the networked epidemiology, the individuals are often referred to as nodes (vertices) and their contacts are referred to as edges. So the constructed social contact network could capture the regular contacts which could lead to the potential transmission of the diseases. Several network models could be candidate contact networks with each leading to different epidemic dynamics. Alternatively, we could incorporate more realistic data into the construction of the contact network with synthetic population generated from the demogeographic information and then organised into different types of groups which are believed to be significant to the transmission of the diseases, such as household group and workgroup [24]. The workplaces have been reported to obey a power law distribution for the number of employees in most countries [15, 25], so do the degree distributions of Portland's social networks [26]. Therefore in our model the individuals in each subpopulation are organised into a scale-free contact network [27, 28].

Let *G*(*V*, *E*) denote the contact graph on a subpopulation *V*; then the edge *e* = (*u*, *v*) of the graph denotes that the individuals *u*, *v* come into contact. For the SEIR model [29] on the network *G*, each node could be in and only in one of the following states at any time *t*: susceptible (*S*), exposed (*E*), infectious (*I*), or removed (*R*). Suppose node *u* is in state *I* at time *t*; then the infection could potentially be transmitted to node *v* along the edge *e* = (*u*, *v*) with a probability *β*_*c*_/*n* at time instant *t* in the discrete-time model, conditional on that *v* remains in *S* until *t* where *n* is the size of the set of neighbours of nodes *v* (denoted by *N*(*v*)). The instantaneous risk of infection *λ*_*c*_ coming from regular contacts for node *v* is defined by

(1) $$\begin{array}{c} \lambda_{c}=\sum_{k\in N(v)}\frac{I_{k}\beta_{c}}{n}. \end{array}$$

Furthermore, if we consider the possible transmission during the random contact within the general community, an addition risk of infection *λ*_*r*_ should be introduced:

(2) $$\begin{array}{c} \lambda_{r}=\sum_{k\in V\setminus N(v)}\frac{I_{k}\beta_{r}}{N-n}. \end{array}$$

So the instantaneous risk of infection for node *v* at time *t* will be

(3) $$\begin{array}{c} \lambda_{v}=\lambda_{c}+\lambda_{r}=\sum_{k\in N(v)}\frac{I_{k}\beta_{c}}{n}+\sum_{k\in V\setminus N(v)}\frac{I_{k}\beta_{r}}{N-n}, \end{array}$$

where *N* is the size of set *V*, *β*_*c*_ and *β*_*r*_ are regular transmission rate and random transmission rate, *V*∖*N*(*v*) denotes individuals belonging to *V* but not belonging to *N*(*v*), and *I*_*k*_ = 1 if individual is infected and 0 otherwise. Therefore the probability of node *v* getting infected at time *t* will be *p* = 1 − exp(−*λ*_*v*_ · Δ*t*).

### 2.2. Travel Behaviours

Although the communities (groups) have been basic components in many studies focusing on the large-scale spatial epidemic dynamics, present researches have simply discarded the long-distance travel behaviours which are greatly associated to these household and work groups. This simplification actually implies that homogeneous mixing assumption is made when quantifying the risk of infection for long-distance travellers; see Figure 1(b). We have introduced the MEs into our model to investigate the influences of the travel types.

Consider a susceptible *j* from *P*_2_ who makes VFR or business travel to the epidemic area (*P*_1_); then *j* will enter into a specific group in *P*_1_, denoted by *C*_1_, as shown in Figure 1(c). In this case, *j* will be more likely infected by individual 2 than other infectives in *P*_1_. Besides, owing to the higher transmission rate within groups, *j* will also suffer from a higher risk of infection. When *j* returns back, it may bring the risk of infection to *P*_2_. Another situation which may put susceptibles in *P*_2_ into risk of infection is that an exposed or infectious individual travels to *P*_2_. As shown in Figure 1(d), infective *i* makes VFR travel to *P*_2_. Instead of leading to an equivalent risk of infection to every individual in *P*_2_, this VFR travel will put individuals in *C*_1_ into a higher risk of infection than other susceptibles. This higher risk will then be transferred to community *C*_2_ through the overlapping individual *k*. Thus the transitivity of the risk will contribute to the dissemination and transmission of the epidemics.

**Figure 2:**
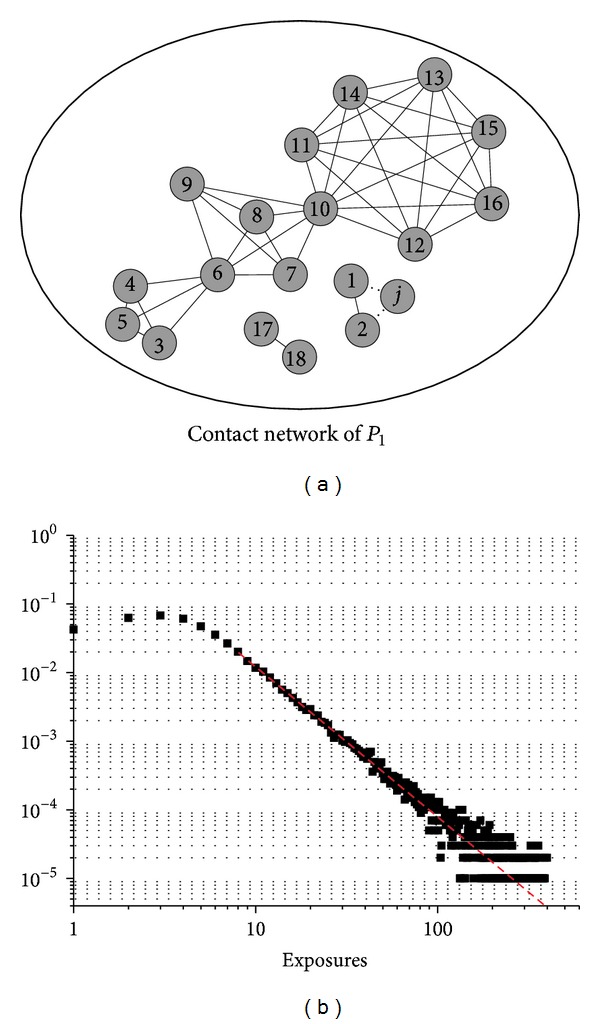
The contact network of the subpopulation. (a) An equivalent contact network to the population structure is shown in Figure 1(c). The long-distance traveller *j* has two high-risk exposures, denoted by node 1 and node 2 and only suffers from occasional risk of infection from other individuals. (b) The distribution of the exposures for individuals in the subpopulations in our model. The dashed red line has a slope −2.19.

Alternatively, the population structure in Figure 1(c) could be described by the contact network in Figure 2(a). The susceptible *j* from *P*_2_ travels to *P*_1_ and enters into group *C*_1_. We could review this process in an equivalent way. Suppose *j* travels to *P*_1_, whose contact network is given in Figure 2(a). If *j* is a business or VFR traveller, the MEs say that it will merge into the contact network of the destination subpopulation, for example, *P*_1_. The MEs take effects in the following two steps: first, *j* randomly selects a node *v* from the contact network of *P*_1_; then all nodes *k* ∈ *N*(*v*) and *v* itself will be added to *N*(*j*). Since the contact network is undirected and reciprocal, *j* will also be in *N*(*k*) for *k* ∈ *N*(*v*) ⋃ {*v*} after this process. In the case of either node 1 or 2 is chosen by *j*, the resulting contact network will be the one in Figure 2(a). However, if *j* is a tourism traveller, it will be regarded as an isolated node in the contact network and only suffers from the risk of infection coming from random contacts in the general community. Notice that large groups tend to absorb more travellers in this definition because they have more group members. Let *ρ* (0 < *ρ* < 1) denote the merging intensity, with larger *ρ* indicating that more travellers will merge into the contact network.

Let *M* be the set of the subpopulations in our metapopulation model and let *P* be the subpopulation which *i* lives in (*i* ∈ *P*). Based on the above analysis, the risk of infection for any susceptible *i* in the presence of long-distance travel types will be

(4) $$\begin{array}{c} \lambda_{i}=\lambda_{\text{IN}}+\sum_{P^{\prime }\in M\setminus \left\{P\right\}}p\left(P,P^{\prime }\right)\cdot \lambda_{\text{EX}}, \end{array}$$

where *λ*_IN_ and *λ*_EX_ are, respectively, the internal and external risks, given by (3), with the network parameters in the equation adjusted according to the contact network which *i* is currently in, and *p*(*P*, *P*′) is the probability that *i* travels to *P*′.

The individuals in subpopulation *P* will make their long-distance travel at the rate *τ*_*P*_ and the long-distance travellers in *P* will end the travel and return to their original subpopulations at the rate *φ*_*P*_.

### 2.3. Model Parameterisation

The reproductive number *R*_0_ is a fundamental parameter for describing the transmissibility of the infectious diseases, which is defined as the average number of secondary infections caused by a typical primary infection in an infinite and completely susceptible population [30]. In structural population, *R*_0_ depends both on the population structure and the characteristics of the virus. We have assumed *R*_0_ = 1.6 according to some recent estimations [15, 21]. Our model involves two transmission rates, for example, *β*_*c*_ from regular contacts and *β*_*r*_ from random contacts. We assume that *β*_*c*_ = 10*β*_*r*_ and the latent and infectious periods are, respectively, 1/*σ* = 1.5 and 1/*γ* = 2.5 days. These parameters integrated with *τ*_*P*_ = 0.0006, *φ*_*P*_ = 0.2, and *ρ* = 0.6 are used to parameterise our model (later we will see that the variation of *τ*, *φ*, and *ρ* does not significantly change *R*_0_) and sensitivity analyses will be made for the primary parameters (*ρ*, *τ*, and *φ*).

In the beginning, both of the subpopulations stay in the fully susceptible state (*S* = 1.0). Then at simulation time *t* = 0, the epidemic is seeded in one subpopulation (*P*_1_). At each time step, a small portion of individuals will make their long-distance travel to another subpopulation or return to their resident subpopulation. The risk of infection for each susceptible is evaluated at each time step (0.5 day).

## 3. Results and Discussion

### 3.1. High-Risk Exposures and the Reproductive Number

In our model, the individuals in each subpopulation are organised into a scale-free contact network. The individual will thus suffer from high risk of infection coming from the daily regular contact with the members within the same group (e.g., neighbours in the contact network). The distribution of the high-risk exposures is shown in Figure 2(b), which displays scale-free regime with *P*(*k*) ~ *k* ^−2.19^, where *k* is the exposures. This structure of the high-risk exposures is consistent with the observations [15, 25, 26].

For compartmental epidemic models, the reproductive number *R*_0_ is determined by the transmission rate *β* and the infectious period 1/*γ* (*R*_0_ = *β*/*γ*), while in structural model, *R*_0_ is determined by the characteristics of both the virus and the structure of the population and the computation is more complicated. An alternative method is to simulate an epidemic and look at the intrinsic growth rate of the infected population [31–34]. This growth rate *r* is defined as the rate at which the total number of infectives, *I*, grows in a susceptible population. The relationship between *R*_0_ and *r* could be derived by the expression of *R*_0_ in terms of the model parameters and the spectral radium of the Jacobian matrix of the dynamic equations, evaluated at *S*_0_ = 1. The SEIR dynamics are defined by the following four differential equations:

(5) S˙=−βSI,E˙=βSI−σE,I˙=σE−γI,R˙=γI.

The corresponding characteristic equation of the Jacobian matrix is

(6) $$\begin{array}{c} \left(r+\sigma \right)\left(r+\gamma \right)=\beta \sigma . \end{array}$$

Therefore

(7) $$\begin{array}{c} R_{0}=\frac{\beta }{\gamma }=\frac{\left(r+\sigma \right)\left(r+\gamma \right)}{\sigma \gamma }. \end{array}$$

**Figure 3:**
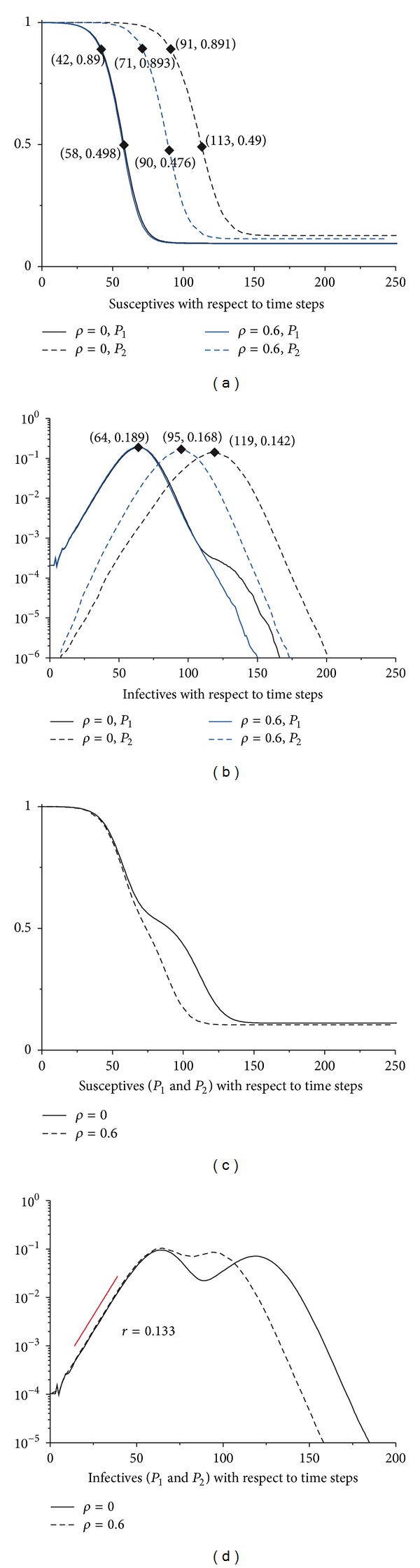
The accelerating and aggravating effects of the MEs. (a) The respective susceptibles in *P*_1_ and *P*_2_ regarding the simulation time steps with no MEs (*ρ* = 0) and merging intensity *ρ* = 0.6. (b) The respective infectives in *P*_1_ and *P*_2_ with respect to the simulation time steps. (c) The aggregated susceptibles in *P*_1_ and *P*_2_ regarding the simulation time steps. (d) The aggregated infectives in *P*_1_ and *P*_2_ with respect to the simulation time steps. For both of the subpopulations, the population size *N* = 1000000, *τ* = 0.0006, and *φ* = 0.2. All the results have been averaged from 100 simulations.

**Figure 4:**
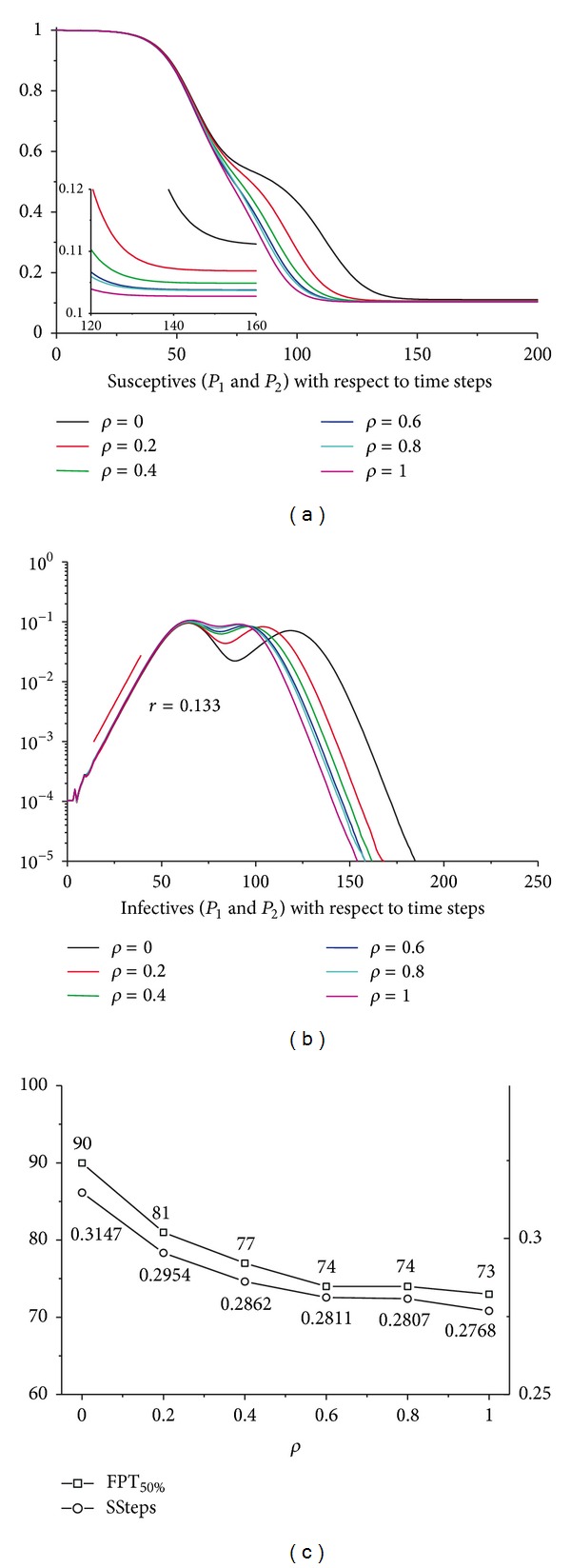
Sensitivity analyses for merging intensity *ρ*. (a) The aggregated susceptibles in *P*_1_ and *P*_2_ regarding the simulation time steps with different *ρ*. The embedded graph is a partial enlargement. (b) The aggregated infectives in *P*_1_ and *P*_2_ with respect to the simulation time steps. (c) The variation of the FPT_50%_ and SSteps(0) for *ρ*. For both of the subpopulations, *N* = 1000000, *τ* = 0.0006, and *φ* = 0.2. All the results have been averaged 100 times.

**Figure 5:**
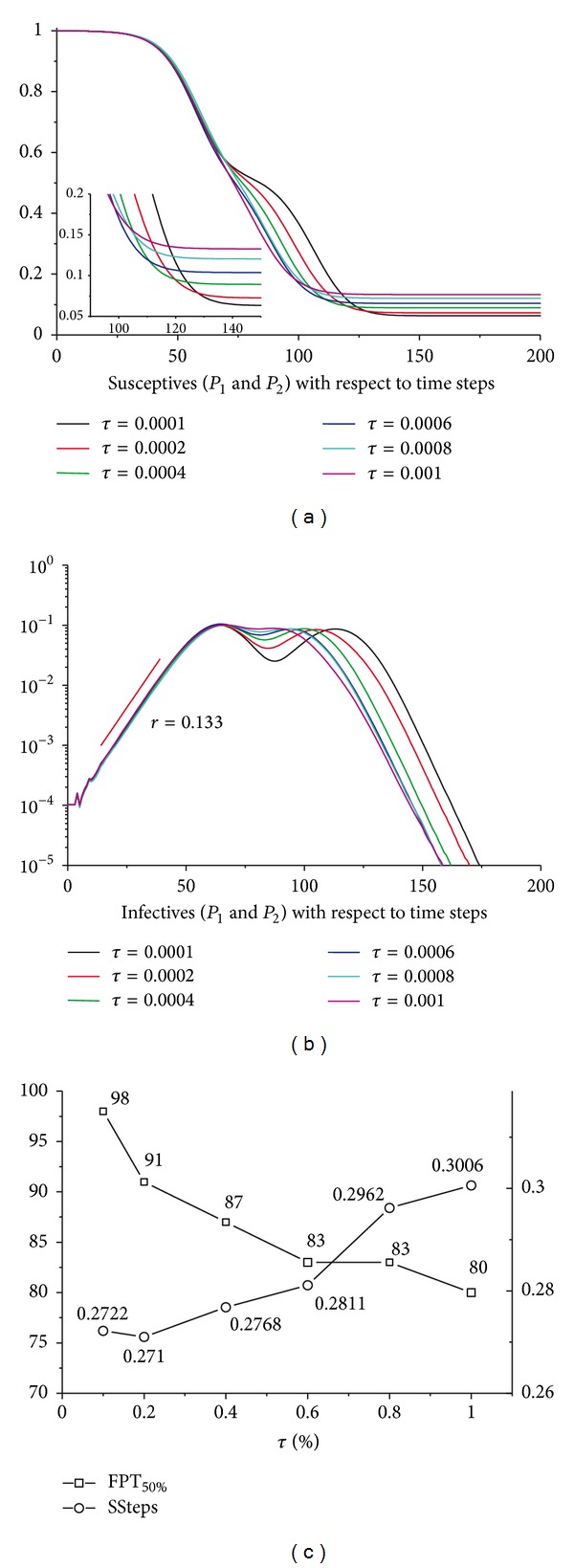
Sensitivity analyses for travel rate *τ*. (a) The aggregated susceptibles in *P*_1_ and *P*_2_ regarding the simulation time steps with different *τ*. The embedded graph is a partial enlargement. (b) The aggregated infectives in *P*_1_ and *P*_2_ with respect to the simulation time steps. (c) The variation of the FPT_50%_ and SSteps(0) for *τ*. For both of the subpopulations, *N* = 1000000, *ρ* = 0.6, and *φ* = 0.2. All the results have been averaged 100 times.

**Figure 6:**
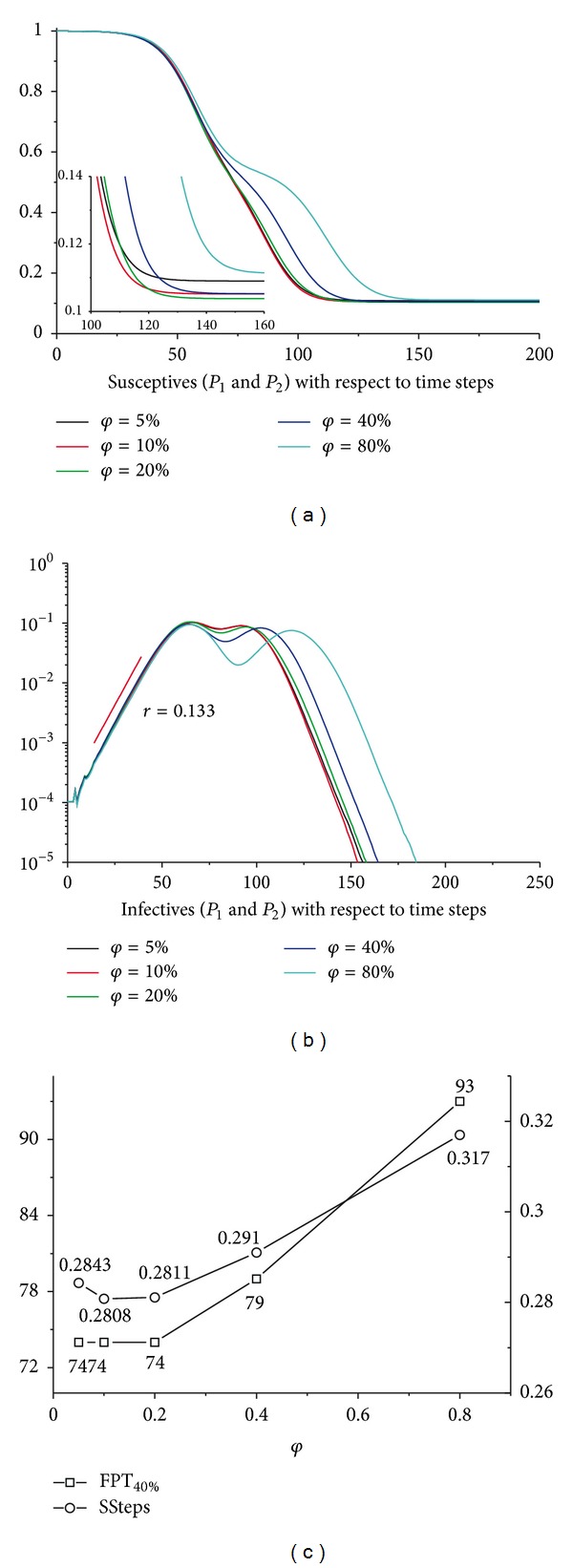
Sensitivity analyses for return rate *φ*. (a) The aggregated susceptibles in *P*_1_ and *P*_2_ regarding the simulation time steps with different *φ*. The embedded graph is a partial enlargement. (b) The aggregated infectives in *P*_1_ and *P*_2_ with respect to the simulation time steps. (c) The variation of the FPT_40%_ and SSteps(0) for *τ*. For both of the subpopulations, *N* = 1000000, *ρ* = 0.6, and *τ* = 0.0006. All the results have been averaged 100 times.

Figure 3(d) shows that in both cases (*ρ* = 0 and *ρ* = 0.6) the intrinsic growth rate *r* = 0.133. Thus we could get *R*_0_ = 1.60. The sensitivity analysis for *ρ*, *τ*, and *φ* indicates that *R*_0_ is relatively constant, as shown in Figures 4(b), 5(b), and 6(b).

### 3.2. Accelerating and Aggravating Effects

We first inspect the accelerating effect of the MEs on the spreading of the epidemics. As shown in Figure 3(a), the susceptibles decrease more rapidly in the presence of the MEs (*ρ* = 0.6). After the initial outbreak at time 0, the susceptibles in the original epidemic stricken population (*P*_1_) will first drop below 90% and 50%, respectively, at time steps 42 and 58 for both scenarios (*ρ* = 0 and *ρ* = 0.6). For population *P*_2_, which is connected to *P*_1_, when *ρ* = 0.6, the susceptibles will first drop below 90% and 50% at time steps 71 and 90, which, respectively, 29 and 32 time steps lagging behind *P*_1_, while in the absence of the MEs (*ρ* = 0), the susceptibles will first drop below 90% (50%) at time step 91 (113). The results indicate that the presence of the MEs has greatly accelerated the spatial spreading process of the epidemics from initial epidemic stricken area (*P*_1_) to the interconnected population (*P*_2_). From Figures 3(b) and 3(c), we could find that the MEs have also aggravated the epidemics in addition to the accelerating effect.

After the evanishment of the epidemics, the individuals in the two populations (*P*_1_ and *P*_2_) which have fortunately escaped from the infection when *ρ* = 0.6 are less than those when *ρ* = 0 (see Figure 3(c)). The investigation on the infectives with respect to the time steps reaches a similar conclusion, which shows that the epidemics will peak much earlier and more dramatically; see Figure 3(b). Another notable outcome is the arising of multipeaks (see Figure 3(d)), which results from the variation in the arrival and peak time of the epidemics in different subpopulations. Figure 3(d) also shows that the intrinsic growth rates in both cases are the same, which indicate that *R*_0_ is the equal.

We then examine the variation of the outcomes when *ρ* varies from 0 (no MEs) to 1 (total MEs).  Figure 4(a) shows the susceptibles in the two populations regarding the time steps with different *ρ*. The accelerating effect and aggravating effect will be strengthened with the increase of the merging intensity. The infectious curves in Figure 4(b) will peak earlier and higher with larger *ρ*, which leads to the same conclusion. To quantify the accelerating effect, here we introduce the concept of the “first passage time (FPT).” Define the *α* (0 < *α* < 1) first passage time of the percentage of individuals in class *A* as the first time step when the percentage of individuals in Class *A* is less than or equal to *α*, denoted by FPT_100*α*%_.  Figure 4(c) shows the variation of FPT_50%_ of the susceptibles of the two populations with different *ρ*. The increase of *ρ* has decreased the FPT_50%_, indicating a more rapid decrease in the susceptibles. The FPT_100*α*%_ is a partial measurement which only involves the information from a single point. A more comprehensive measurement is the susceptible steps analogous to person years or person months which are widely used when performing certain types of prospective studies—studies following a large group of people over time. The person years or person months measurement takes into account both the number of people in the study and the amount of time each person spends in the study. Define the susceptible steps (SSteps) as the aggregate normalised susceptibles over the concerned time period as follows (for the continuous scenario, we could simply change the summation into the integration):

(8) $$\begin{array}{c} \text{SSteps}\left(t_{0}\right)=\frac{1}{T-t_{0}}\sum_{t=t_{0}}^{T}S_{t}, \end{array}$$

where *T* is the simulation period and *S*_*t*_ denotes the susceptibles at time step *t*. Equation (8) indicates that the SSteps(*t*) are actually the areas surrounded by the axes, the susceptible curve, current time step *t*, and the simulation period *T*. Notice that the SSteps(*t*) defined above have measured both the accelerating effect and the aggravating effect. A more rapid drop of the susceptibles and a lower escape rate both will decrease the SSteps(*t*). The sensitivity analyses show that the SSteps at time *t* = 0 decrease with the increase of *ρ* (see Figure 4(c)), which indicates that the presence of the MEs has greatly accelerated and aggravated the epidemics.

### 3.3. Travel Rate and Return Rate

The travel rate (*τ*) and return rate (*φ*) are two important parameters in our model. The travel rate has dominated the closeness between the interconnected subpopulations. A higher travel rate implies that there are more daily long-distance travellers between these interconnected subpopulations.  Figure 5 shows the sensitivity analyses for *τ* ranging from 0.0001 to 0.001. We could find that the increase of the travel rate has accelerated the spatial pervasion of the epidemics, while it also leads to a higher escape rate (see Figures 5(a) and 5(c)).

The return rate will affect the transmission dynamics of infectious diseases in two ways. First, the return rate has an effect on the individual mobility between the interconnected subpopulations. A higher return rate implies that more individuals will return back to their resident subpopulations and thus refers to a higher human mobility. On the other hand, a higher return rate means that the duration of the long-distance travel (with mean duration 1/*φ*) will be much shorter and thus will decrease the probability of being infected and of infecting the others. So the aggregate influence of the return rate is uncertain, which is verified by Figure 6.

### 3.4. Metapopulation Simulation

**Figure 7:**
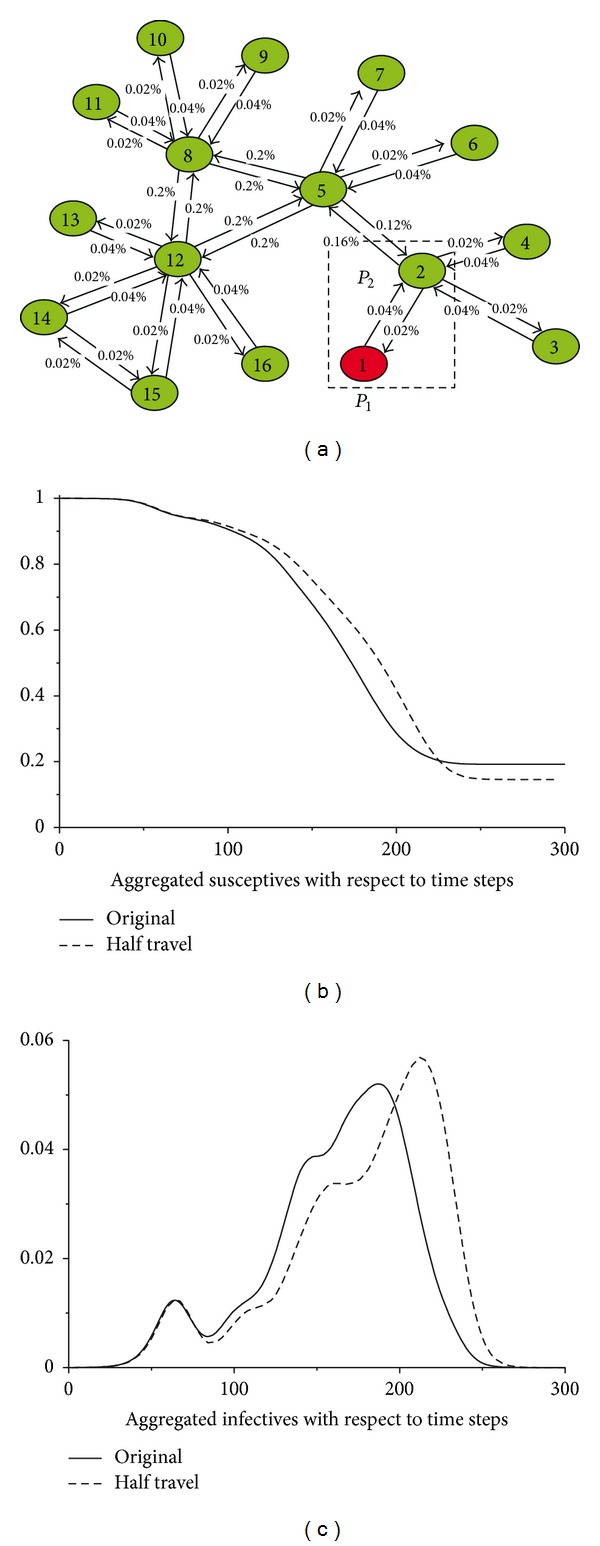
The Metapopulation simulation. (a) The mobility network with *τ* noted on the corresponding link. (b) The aggregated susceptibles with respect to the time steps. (c) The aggregated infectives regarding the time steps. For all of the subpopulations, *N* = 1000000, *ρ* = 0.6, and *φ* = 0.2. The results have been averaged 20 times.

To illustrate the influences of the MEs on the large-scale spatial disease dynamics, we have conducted a simulation which involves more subpopulations; see Figure 7(a). The mobility network is exactly the same as shown in Figure 1(a) except that the travel rates have been marked on each link. Notice that we apply different *τ* for these routes. This is reasonable because of the heterogeneity in real human mobility patterns [35]. In the beginning, all the subpopulations stay susceptible (*S* = 1.0). At the time *t* = 0, the epidemic is seeded into *P*_1_. The population structure and the MEs assumption are exactly the same as we discussed above.

In Figures 7(b) and 7(c), we show the aggregated susceptibles and infectives (solid lines) with respect to the time steps. Both of the epidemic curves have fluctuated obviously due to the variation in the arrival and peak time of the epidemics in different subpopulations. We also investigate the situation that every *τ* on the link halves. The results are figured by the dashed lines in Figures 7(b) and 7(c). We could find that the lower travel flows have slowed down the diffusion of the diseases, while leading to more infectives.

## 4. Conclusion

In this paper, we have characterised the influences of long-distance travel behaviours on the large-scale spatial disease dynamics. The results indicate that the presence of the MEs has greatly speeded up the transmission of the diseases and also aggravated the epidemics. The sensitivity analyses for the merging intensity have shown the constant presence of the accelerating and aggravating effects, which are even stronger with large merging intensity. We also find that a higher travel rate will contribute to the rapid transmission of the diseases, while it will alleviate the outbreaks. The influences of the return rate are relatively uncertain due to its duplicity.

Our research has highlighted several important causes which could significantly affect the large-scale spatial spreading of infectious diseases in addition to the fully explored parameters such as the transmission rates within and between different groups, mainly characterised by the merging intensity (which is related to the travel structure), the travel rate, and the return rate. These highlighted parameters should be taken as important focuses of further studies. And also survey is required for a deeper understanding of long-distance travel behaviours.
